# Consumption of Yogurt in Canada and Its Contribution to Nutrient Intake and Diet Quality Among Canadians

**DOI:** 10.3390/nu11061203

**Published:** 2019-05-28

**Authors:** Hassan Vatanparast, Naorin Islam, Rashmi Prakash Patil, Arash Shamloo, Pardis Keshavarz, Jessica Smith, Susan Whiting

**Affiliations:** 1 College of Pharmacy and Nutrition, School of Public Health, University of Saskatchewan, Saskatoon, SK S7N 4Z2, Canada; naorin.islam@usask.ca (N.I.); rashmi.patil@usask.ca (R.P.P.); arash.shamloo@usask.ca (A.S.); pak526@mail.usask.ca (P.K.); susan.whiting@usask.ca (S.W.); 2 Bell Institute of Health and Nutrition, General Mills, Minneapolis, MN 55427-3870, USA; Jessica.Smith@genmills.com

**Keywords:** yogurt, nutrient intake, dietary assessment, diet quality

## Abstract

The current study utilizes a nationally representative nutrition survey data (Canadian Community Health Survey 2015, nutrition component, *n* = 20,487) in order to evaluate patterns of yogurt consumption among Canadians. Overall, 20% of Canadians have reportedly consumed yogurt on a given day in 2015. Higher prevalence of yogurt consumption was noted among children aged 2–5 years old (47%) when compared to adults aged 19–54 years (18%). When the prevalence of yogurt consumption at the regional level in Canada was assessed, Quebec had the most consumers of yogurt (25%) compared to other regions, namely the Atlantic (19%), Ontario (18%), Prairies (19%) and British Columbia (20%). Yogurt consumers reported consuming higher daily intakes of several key nutrients including carbohydrates, fibre, riboflavin, vitamin C, folate, vitamin D, potassium, iron, magnesium, and calcium when compared to yogurt non-consumers. Additionally, the diet quality, measured using NRF 9.3 scoring method, was higher among yogurt consumers compared to non-consumers. Nearly 36% of Canadians who meet the dietary guidelines for milk and alternative servings from the Food Guide Canada (2007) reported consuming yogurt. Lastly, no significant difference in BMI was noted among yogurt consumers and non-consumers. Overall, yogurt consumers had a higher intake of key nutrients and had a better diet quality.

## 1. Background

According to the FDA, yogurt is defined as the “food produced by culturing one or more of the optional dairy ingredients (cream, milk, partially skimmed milk, and skim milk) with a characterizing bacteria culture that contains the lactic acid-producing bacteria” [1]. Before adding flavours, yogurt contains at least 3.25% milkfat, 8.25% milk solids not fat, and 0.9% lactic acid for the acidity. On the other hand, low-fat yogurt and nonfat yogurt contains 0.5–2% and less than 0.5% milk fat respectively [2]. Another definition from The FAO/WHO Codex Alimentarius Commission defines yogurt as “a coagulated milk product obtained by lactic acid fermentation through the action of *Lb. delbrueckii* subsp. *bulgaricus* and *St. thermophilus* from milk (pasteurized or concentrated milk) with or without additions (milk powder, skim milk powder, etc.)”. The ultimate product contains plentiful and viable microorganisms [3,4].

The consumption of yogurt has been steadily increasing over the past ten years in Canada. From 2007 to 2016, average yogurt consumption increased by 43.7%, from 7.66 litres to 11.01 litres per capita. No mandatory fortification is required for yogurt in Canada [5]. Fortified yogurt stays one of the most popular dairy products in Canada due to an increase interest of consumers for ingredients such as probiotics. Drinkable yogurt is also becoming gradually favoured among Canadians [6]. Yogurt was reported as consumed in the Canadian household approximately 2–3 times per week in 2004. Approximately 70% purchase either fat-free or low-fat yogurt and over 80% purchase flavoured yogurt [7]. Americans could meet the recommended intake for dairy with help of yogurt since it has been identified as a nutrient-dense food [8], and cross-sectional studies have shown that yogurt intake is associated with better diet quality [9].

The impact of yogurt on obesity and weight management has been studied through observational and experimental studies, with few studies focused specifically on children [9]. In Canada, to our knowledge, there is no comprehensive national study evaluating the role of yogurt in the Canadian diet. The purpose of this study was to use the recent nationally representative nutrition survey, Canadian Community Health Survey (CCHS) 2015, to evaluate yogurt consumption pattern, the contribution of yogurt to nutrient intakes, and the association with diet quality (measured using the nutrient rich foods index 9.3 applied to the total diet), and health status of Canadians by age, sex, region of residence, and socioeconomic status, in order. 

## 2. Subject and Method

### 2.1. Data Source and Analytical Sample

#### 2.1.1. Dietary Data Collection

The current study is based on the results from the Canadian Community Health Survey data (CCHS) Nutrition 2015. It is a cross-sectional survey conducted by Statistics Canada. The target population for the survey included individuals from 10 Canadian provinces who were one year old and above living in private dwellings. The survey was based on a 24 h dietary recalls where respondents were asked about their food and beverage consumption and sociodemographic status such as age, sex, province of residence, immigration status, BMI, food security status, smoking status, ethnicity, marital status, overweight/obesity, education status. The final sample size of the first recall was 20,487 representing 98% of the population. 

Dietary data was collected via computer-assisted personal interview using the Automated Multiple Pass Method (AMPM) from respondents for day 1 of 24-h recall, and a telephonic interview for day 2 of 24-h recall. The current study uses only the day 1 of 24-h recall. For children aged 1 to 6 years, proxy interviews were carried out. Children aged 6 to 12 years participated in the interview with parental guidance, and individuals above 12 years were interviewed by the non-proxy method. This survey used Canadian Nutrient File 2015, which includes nutrient profile of food items in Canadian market, for coding dietary intake data [10]. Detailed information on sampling and CCHS 2015 Nutrition survey can be found elsewhere [10]. Permission to access the CCHS 2015 data and conduct analysis was obtained from the Research Data Center Program of Statistics Canada. 

#### 2.1.2. Analytical Sample

This study included 19,677 respondents representing Canadian population who were two years of age and older after excluding pregnant, lactating women, individuals who did not report any food in the 24-h recall, and individuals with <200 kcal and >8000 kcal energy intake [11]. Individuals with extreme high intakes of nutrients and food group were excluded from the study. Yogurt consumer was defined as any individual who has reported consuming any serving (>0 serving) of yogurt food codes on day 1 of 24-h recall. In this study, 4072 individuals reported yogurt consumption. The yogurt food codes were derived from the Canadian Nutrient File and frozen yogurts were not included in this study. We have included several types of yogurt including plain yogurt, coffee, fruit, vanilla falovoured yogurt, greek yogurt, goat yogurt, soy yogurt and yogurt beverages. Prevalence of yogurt consumption has been summarized by five age groups (2–5 years, 6–12 years, 13–18 years, 19–54 years and 55+ years), five regions (Atlantic, Quebec, Ontario, Prairie and British Columbia). Sociodemographic differences between yogurt consumers and non-consumers had been reported by two age groups (children: 2–18 years, adult: 18+ years). The frequency of yogurt consumption at different food occasions such as breakfast, snack, lunch, dinner was reported.

#### 2.1.3. Descriptive Characteristics

Several descriptive characteristics by age and yogurt consumption status including sex, smoking, education, marital status, food security, physical activity (self-reported), overweight/obese, residence (urban, rural), and immigrant were reported. For region-specific analyses, the Canadian provinces were merged into five regions including Atlantic (Newfoundland and Labrador, Nova Scotia, New Brunswick), Ontario, Quebec, Prairies (Manitoba, Saskatchewan, and Alberta), and British Columbia. Sociodemographic. BMI z-score for children age 5 to 18 years was calculated following the protocol from the World Health Organization (WHO) [12]. Measured BMI values for adults is available in CCHS 2015 which had been used in this study. Sociodemographic variables were categorized as follows: sex (male, female), smoking (yes, no), ethnicity (Caucasian, non-Caucasian), education (university degree, lower than university degree), marital status (yes, no), food security (secure, insecure), overweight/obese (yes, no), physical acitivity (yes, no), residence (urban, rural), immigrant (yes, no) and age (2–5 years, 6–12 years, 13–18 years, 19–54 years and 55+ years). The percentages of individuals who are meeting the dietary recommended serving sizes of milk and alternatives had been calculated among yogurt consumers and non-consumers. The recommended serving sizes of milk and alternatives had been derived from the Canada Food Guide, 2007 [13].

#### 2.1.4. Daily Nutrient and Energy Intake

Mean energy and nutrient intakes were reported for both yogurt consumers and non-consumers. The contribution of yogurt to daily nutrients intake was also determined for all yogurt consumers as five age groups (2–5 years (*n* = 545), 6–12 years (*n* = 640), 13–18 years (*n* = 400), 19–54 years (*n* = 1259) and 55+ years (*n* = 1228)).

#### 2.1.5. Nutrient Density

Diet quality of yogurt consumers and non-consumers had been measured using The Nutrient-Rich Foods Index 9.3 which is an indicator to estimate the nutrient density of the overall diet. This index is calculated by taking the sum of the percent of daily values of nine nutrients known as nutrients to encourage (protein, fibre, vitamin A, vitamin C, vitamin E, calcium, iron, magnesium, and potassium) minus the sum of percent of the maximum recommended values of three nutrients, known as nutrients to limit (saturated fat, added sugar and sodium) [14].

We slightly modified the NRF scoring procedures by replacing added sugars and vitamin E by total sugar and vitamin D because CCHS 2015 does not contain any information on added sugar or vitamin E [11]. For the nine nutrients to encourage, percent daily values of each nutrient were truncated at 100 if the intakes exceeded the daily values. Percent daily values for three nutrients to limit were taken as zero if the intake is less than daily values [11]. The maximum possible NRF score is 900, which is possible if an individual’s total intake in a day is 2000 kcal and they meet or exceed the daily value for the nine nutrients to encourage and are less than the daily value for the three nutrients to limit [11]. Higher NRF score indicates better diet quality. The daily values’ were based on recently updated Canadian DV’s for nutrient intakes [15]. 

### 2.2. Statistical Analyses 

SAS software, version 9.4, used to perform all the analyses for this study. Statistics Canada’s recommended weighting and bootstrapping were applied to all the analyses to produce a population-level estimate including frequencies [16]. For continuous variable analyses, the values are represented in means and standard errors, and for categorical variable analyses, the values are represented as percentages and standard errors. For comparing the distribution of categorical sociodemographic variables between yogurt consumers and non-consumers, the chi-square test was used. We used the Student’s t-test for comparing the distribution of continuous variables between yogurt consumers and non-consumers. Variables that differed significantly between yogurt consumers and non-consumers were used as covariates in regression models comparing daily energy and nutrient intakes. Analysis of covariance (ANCOVA) statistical method was used to determine the differences across age groups in mean daily energy intake, daily nutrient intakes and nutrient contribution of yogurt to mean daily energy and nutrients. To obtain differences of daily energy intake, daily nutrient intake and proportion of nutrient contribution between yogurt consumers and non-consumers similar test was applied. P-value had been set to 0.05 for statistical significance in all analyses.

## 3. Results

Overall 20% of Canadians reported consuming yogurt on any given day. The prevalence of yogurt consumption was significantly different across five age groups and children reported higher consumption (2–5 years: 46.9% ± 2.5%, 6–12 years: 29.5% ± 1.6%) when compared to adolescents (13–18 years 14% ± 0.9%), adults (19–54 years 18.4% ± 1%) and older adults (≥55 years 19.1% ± 0.9%). A higher proportion of females consumed yogurt than males (23.5% ± 0.8% vs. 16.9% ± 0.8%), respectively). Almost 37% ± 1.9% of individuals among yogurt consumers reportedly consume yogurt at a snack occasion, followed by 32% ± 1.3% of individuals consuming at breakfast, 25.5% ± 2.4% at lunch, and 13.9 ± 0.8% at dinner. The standard serving size for yogurt is 175 gram. Overall, the mean serving size of yogurt consumtion was 0.77 ± 0.02 in a day for Canadians aged 2 years and over. Moreover, children (2–5 years: 0.79 ± 0.03, 6–12 years: 0.84 ± 0.04) had reported higher serving sizes of yougurt consumption than adolescents (13–18 years: 0.75 ± 0.04), adults (19–54 years: 0.75 ± 0.03) and older adults (≥55 years: 0.76 ± 0.03) in a day. Among yogurt consumers, 36.0% ± 1.6 met or exceeded the daily recommended serving guidelines for milk and milk alternatives based on the Canada Food Guide (2007) [13]. Conversely, only 16.0 ± 1.6 of yogurt non-consumers met those guidelines.

### 3.1. Prevalence of Yogurt Consumption at National Level 

Table 1 represents variations in socio-demographic characters of yogurt consumers and non-consumers. Yogurt consumers among children/teens are significantly younger. They more likely have a university degree in the household, are food secure and are less likely to be males, overweight/obese or immigrants. Among adults, yogurt consumers were significantly older, more likely to be married, be food secure, be of Caucasian race, have a university degree, and less likely to smoke and be a male. 

### 3.2. Prevalence of Yogurt Consumption at Regional Level 

The percentages of yogurt consumers across sex and age groups are presented for the five Canadian regions in Table 2. Quebec had the highest number of yogurt consumers with 25.4% ± 1.5% when compared to other regions/provinces ranging between 17.9% ± 1% in Ontario to 20% ± 1.2% in British Columbia. Among children aged 2–18 years, the percent of yogurt consumers was the highest in Quebec at 31.6% ± 2.1% with the lowest being in the Atlantic region at 20.2% ± 1.5%. Among adults aged ≥19 years, the percent of consumers were the highest in Quebec with 24.1% ± 1.7% and lowest for Ontario at 15.9% ± 1.2%. 

### 3.3. Daily Energy and Nutrient Intake Comparison between Yogurt Consumers and Non-Consumers 

Table 3 exhibits mean energy and nutrient intakes of yogurt consumers and non-consumers for the Canadian population (≥2 years) and by five age groups. For all Canadians yogurt consumers, intake of carbohydrates and dietary fibre was significantly higher in yogurt consumers in comparison to non-consumers. Vitamin and mineral intakes of riboflavin, vitamin C, folate, vitamin D, potassium, iron, magnesium and calcium were significantly higher in yogurt consumers. Similar patterns of intake were seen for five age groups and can be seen in Table 3.

### 3.4. The Contribution of Energy and Nutrients from Yogurt to Daily Nutrient Intake

Table 4 displays the percentage nutrient contribution of yogurt to daily nutrient intake for Canadians aged ≥2 years, and for five age groups. For Canadians who reported eating yogurt, yogurt contributed to nearly 5.7% to daily energy intake among yogurt consumers. Nutrients such as protein, vitamin A, vitamin D, riboflavin, vitamin B_12_, calcium, and potassium proportionally contributed more to daily nutrients compared to energy intake. The contribution towards daily energy intake among children 2–5 years was 6.8%, 6–12 years was 5.7%, adolescents 13–18 years was 5.1%, adults 19–54 years was 5.4% and older adults’ ≥55 years was 5.7%. Similar patterns to those of all Canadians were seen for age group breakdown when observed for nutrient contributions. 

### 3.5. Differences in Nutrient Density between Yogurt Consumers and Non-Consumers

Table 5 shows the difference in NRF 9.3 scores between yogurt consumers and non-consumers at the national level (all ages and five age groups), and across regions (all ages and two age groups). NRF 9.3 score was significantly higher across yogurt consumers than non-consumers. At the national level older adults, aged ≥55 years had the highest NRF 9.3 among yogurt consumers when compared to non-consumers (578.8 ± 5.1 vs. 528.7 ± 3.3) respectively. At the regional level, British Columbia had the highest NRF 9.3 score among adults (582.2 ± 10.9 vs. 517.7 ± 6.3) and children (552.1 ± 9.8 vs. 515.1 ± 6.6) than other regions. 

### 3.6. Differences in BMI between Yogurt Consumers and Non-Consumers

Yogurt consumers and non-consumers did not have significant differences in their mean BMI both for children and for adults. Among children (2–18 years) the BMI z-score for yogurt consumers was 0.46 ± 0.1 and non-consumers was 0.47 ± 0.01 without significant difference. For adults ≥19 years, the mean BMI for yogurt consumers was 27.1 ± 0.2, while it was 27.4 ± 0.1 for none-consumers, with no significant difference. We also found no significant differences in physical activity between yogurt consumers and non-consumers. Among consumers, 70% (95% confidence interval: 67, 73) reported participating in physical activity and among non-consumers, 68% (95% confidence interval: 66, 70) reported participating in physical activity for age ≥2 years.

## 4. Discussion 

To our knowledge, this is the first study to comprehensively evaluate the role of yogurt in Canadians’ nutrient intakes, and examine the NRF 9.3 score for yogurt consumers vs. non-consumers. We found the diet of yogurt consumers had higher nutrient density than non-consumers. The overall rate of yogurt consumption among Canadians was 20%, of which 2–5 year-old children had the highest consumption compared to other age groups in the study. Results indicate that yogurt consumption was associated with higher intake of key nutrients. Some of these differences in the diets of yogurt consumers can be attributed to the yogurt itself but also reflect the overall healthier diets of yogurt consumers. Furthermore, there were no significant differences between BMI among yogurt consumers and non-consumers.

Overall, one in five Canadians are yogurt consumers and children had the highest proportion of yogurt consumers. Using data from the National Health and Nutrition Examination Survey (NHANES) 2005–2008, Keast et al. reported that among children and adolescents aged 8–18 years, only 8.5% were yogurt consumer [17]. Another study which used CCHS 2.2 (2004) data reported that less than 1% of respondents consumed yogurt [7]. Both of these studies reported numbers varying greatly from our study. This increase in the proportion of yogurt consumption may be due to the availability of a variety of yogurt products and the rising demand of consumers [18]. Another national survey in the UK also reported that children aged three years and under had the highest intakes of yogurt compared to adults which were equivalent to less than a third of a standard [19]. Furthermore, in our study, females had a higher tendency to consume yogurt than males which was similarly reported in Wardle et al [20]. This can be due to gender differences in food choices reflecting women’s greater tendency towards weight control and partially to their perceptions of healthy eating.

Our findings indicated that the highest proportion of yogurt consumption occurs in Quebec and this region also has the highest percentage among 2–18 year-old children. In Canada, West et al. (2002) found that consumers with children in the household, men, metropolitan consumers, and consumers in Quebec were the most willing to purchase functional foods such as probiotic yogurts which verifies our results in terms of age group and province [21]. We found no other studies which evaluate the consumption of yogurt in Canada. 

In our study, yogurt consumption was associated with higher intake of carbohydrate, dietary fibre, vitamin B_12_, vitamin C, folate, vitamin D, potassium, magnesium, total sugar (which was statistically significant among who aged >55 years old) and calcium. On the other hand, energy (kcal/day, and total fat (g/day) did not differ between consumers and non-consumers. These results are similar to those reported by Keast et al. [17] who reported yogurt and dairy are common sources of calcium and vitamin D and some other key nutrients. However, the overall healthy dietary pattern of yogurt/dairy consumers also lead to the positive associations between intake of these foods and consumption of these nutrients. While the U.S. Department of Agriculture (2010) reported greater overall dairy consumption has been associated with higher energy intake along with higher saturated fat intake (as % of daily energy) [22], we did not find higher energy or saturated fat intake specifically from yogurt consumption. 

The contribution of nutrients from yogurt among yogurt consumers provides higher nutrient density. Yogurt contributed approximately, 6% of daily energy intake and was an important source of protein, vitamin D, riboflavin, vitamin B_12,_ calcium, potassium, saturated fat and total sugar in the diets of yogurt consumers but did not contribute greatly to fibre, thiamin, vitamin C, folate, iron, magnesium or fat intake. Yogurt was a particularly important source of protein, vitamin D, vitamin B_12_, and calcium for people more than 55 years old. In the UK, a study presented the contribution of yogurt to energy and nutrient intakes across the life course through secondary analysis of data from the Diet and Nutrition Survey (2011) which showed a similar pattern of results as we found our study [19].

We found that yogurt consumers were more likely to meet the recommended number of servings of milk and alternatives per day than yogurt non-consumers (based on the recommended number of servings on the Canada Food Guide (2007) [13]) meaning that yogurt consumers are eating yogurt as a snack more than non-consumers. It is noteable that the recently released Canada Food Guide (2019) dose not quanity the recommended intake based on serving sizes or gram intake for all food groups [23]. Based on these findings, however, due to the important contribution of yogurt to nutrient intake and diet quality, consuming low sugar and low fat probiotic yogurt among Canadians can be encouraged while barriers to accessibility, culture and cost are resolved. 

One way of assessing the nutritional quality of foods is through nutrient profiling which involves the use of scientific ranking or classification of foods based on their nutrient compositions. Several nutrient profiling models have been developed including the NRF index score to reflect the nutrient quality of foods. According to the 2015 Dietary Guidelines for Americans [24], nutrient density refers to “a characteristic of foods and beverages that provide vitamins, minerals, and other substances that contribute to adequate nutrient intakes or may have positive health effects, with little or no solid fats and added sugars, refined starches, and sodium. Ideally, these foods and beverages also are in forms that retain naturally occurring components, such as dietary fibre. All vegetables, fruit, whole grains, seafood, eggs, beans and peas, unsalted nuts and seeds, fat-free and low-fat dairy products, and lean meats and poultry-when prepared with little or no added solid fats, sugars, refined starches, and sodium- are nutrient-dense foods”. Generally, nutrient dense foods provide a substantial amount of vitamins and minerals (micronutrients) but relatively few calories [14]. We used the nutrient density of the overall diet as a measure of diet quality using the NRF 9.3 method. NRF scores between yogurt consumers (higher score) and non-consumers depicted that yogurt consumers had higher diet quality when compared to non-consumers at all population and age breakdown levels. Yogurt consuming adults aged ≥55 years had the highest score and it can be, in part, related to the high contribution from yogurt to daily nutrients intake in this age group. At the provincial level, adults in British Columbia had the highest score. British Columbia was also the region with the second highest prevalence of yogurt consumption, after Quebec. 

We found no significant differences in BMI or BMI z-score (for children) between yogurt consumers and non-consumers. Some previous research had found an inverse association between yogurt consumption and weight gain [9,25] and there are several proposed mechanisms by which yogurt could play a role in weight maintenance. However, other studies found similar results to our findings [17,26]. Zhu et al., using the NHANES 2003–2006 survey among 2–18 years old children, found that the body weight changes between frequent yogurt consumers and infrequent consumers were not significant [26]. In another study, Keast et al. using NHANES 2005–2008, among children 8–18 years reported the differences in terms of body weight were not significant, yogurt consumption was associated with lower BMI [17]. However, no evidence has been reported to positively associate yogurt with weight gain. Whether yogurt may aid in weight loss or weight gain prevention requires further research is warranted considering differences in yogurt composition including different texture, fat proportion, and culture bacteria composition.

Yogurt can be considered as a healthy snack compared to other options. A greater reduction in appetite was observed with the yogurt snack compared to the cheese and milk snacks [27]. Since the majority of snacking in the United States takes place in the afternoon and evening, rather than the morning, a study was conducted in the US among fifteen healthy women (age: 26 ± 2 y) that randomly consumed 160 kcal afternoon yogurt snacks containing low, moderate, or high protein (5, 14, or 24 g protein, respectively) or had no snack (NS) for three days. The results showed that the consumption of a 160 kcal afternoon yogurt snack, varying in protein content, led to reduced afternoon hunger and delayed the onset of eating. This effect was higher with high protein yogurt. These data indicate that a small portion, high protein afternoon yogurt snack regimen might delay or prevent subsequent over-eating later in the day [28]. In our study, we found 37% of yogurt consumers eat yogurt as a snack and this eating behaviour should be promoted in Canada in order to obtain a healthier eating pattern.

### Strengths and Limitation

The current study used CCHS 2015 Nutrition data, which is a recent national level comprehensive nutrition and health study, representing all Canadians. Implausible reporting of nutrient intake based on energy and food group consumption were identified at data cleaning stages and were removed from the study. Other strengths of the study include high-quality dietary assessment method (AMPM); using measured BMI (not self-reported); adjusting for key confounding variables; reporting regional data, in addition to national level, which is important in a geographically large and diverse nation. However, there are some limitations as well. The study is based on cross-sectional data, hence no causal inference can be made based on our results. There can be errors and biases in self-reported dietary intake data. This study is based on day 1 of 24 h recall which may not indicate usual food intake along with weekend food consumption. The mean intake of day 1 and day 2 are approximately the same in survey data, although using the consecutive days of 24-h recall provide more robust usual intake estimates. The original NRF 9.3 method for diet quality assessment uses data on vitamin E. However, we replaced vitamin E with vitamin D in the absence of vitamin E intake data. 

## 5. Conclusions

Nearly, one-fifth of Canadians reported consuming yogurt on any given day in 2015. Compared to non-consumers, yogurt consumers had a healthier nutrient intake profile and better diet quality. Compared to the other popular snack items, encouraging the consumption of low sugar and low fat yogurt as a snack may provide a healthier diet. 

## Figures and Tables

**Table 1:** Sociodemographic characteristics of Canadians children and adults among yogurt consumers and non-consumers ^1^.

Characteristic	Children/Teens (2–18 years) (*n* = 6,429,627)		Adults (≥19 years) (*n* = 27,006,075)	
	Yogurt Consumers (*n* = 1,704,953)	Yogurt Non-Consumers (*n* = 4,724,674)	Yogurt Consumers (*n* = 5,043,410)	Yogurt Non-Consumers (*n* = 21,962,665)
Mean age +/- SD (year)	7.7 ± 0.1	10.7 ± 0.0 *	50.0 ± 0.6	49.2 ± 0.2 *
Sex (% male)	46.9 ± 1.8	51.3 ± 0.9 *	39.8 ± 1.8	52.1 ± 0.4 *
Smoker (% yes) ^2^	1.7 ± 0.7	3.8 ± 0.5	11.7 ± 1.3	20.4 ± 0.8 *
Ethnicity (% Caucasian)	70.2 ± 2.1	66.4 ± 1.0	78.5 ± 1.7	74.2 ± 1.0 *
Education (% university grad) ^3^	48.6 ± 2.0	42.9 ± 1.4 *	45.8 ± 2.1	36.6 ± 0.9 *
Marital status (% married or co-habiting)	n/a	n/a	69.9 ± 1.7	62.6 ± 0.9 *
Food secure (% yes)	89.0 ± 1.3	81.2 ± 0.9 *	92.4 ± 1.0	87.6 ± 0.6 *
BMI (kg/m^2^)	n/a	n/a	27.1 ± 0.2	27.41 ± 0.1
BMI z-score ^4^	0.45 ± 0.0	0.5 ± 0.0	n/a	n/a
Overweight/obese (% yes)	21.8 ± 1.6	27.8 ± 1.3 *	61.5 ± 2.0	61.9 ± 1.1
Urban residence (% yes)	82.2 ± 1.1	82.2 ± 1.6	80.7 ± 1.6	82.9 ± 0.8
Immigrant to Canada (% yes)	6.8 ± 1.0	9.8 ± 0.7 *	25.4 ± 1.8	27.8 ± 1.0

Data source: 2015 Canadian Community Health Survey—Nutrition. Yogurt consumers were defined as those individuals reporting any quantity of yogurt consumption on their day 1 of 24-h recall. * Significant at 0.05 level of significance using chi -squared test for categorical variables and t-test for continuous variables. Yogurt consumers were compared to yogurt non-consumers separately for children and adults. ^1.^ All data are weighted and bootstrapped to obtain estimates at the Canadian population level. ^2.^ Smoking data was only available for children aged ≥ 12 y. ^3.^ For children, the variables reflects whether an adult member of the household is a university graduate. ^4.^ For those age 5–18 years, based on BMI z-score for age and sex.

**Table 2:** Distribution of yogurt consumers by Canadian regions, age and sex groups ^1^.

Characteristics	Regions				
	Atlantic (*n* = 409,046)	Quebec (*n* = 1,988,464)	Ontario (*n* = 2,327,589)	Prairies (*n* = 1,142,808)	British Columbia (*n* = 880,456)
Yogurt consumers (% ± SE)	18.6 ± 0.9	25.5 ± 1.5	17.9 ± 1.0	19.01 ± 1.0	20.0 ± 1.2 *
Yogurt consumers					
Male (% yogurt consumers ± SE)	13.1 ± 1.1	20.6 ± 2	15.7 ± 1.4	15.7 ± 1.4	16.9 ± 1.6
Female (% yogurt consumers ± SE)	23.6 ± 1.4 **	30.4 ± 2.3 **	20.0 ± 1.3 **	22.4 ± 1.5 **	23.2 ± 1.9 **
Yogurt consumers					
Age 2–18 years yogurt consumers (% ± SE)	20.2 ± 1.5	31.6 ± 2.1	26.0 ± 1.7	24.0 ± 1.4	26.2 ± 2
Age ≥19 years yogurt consumers (% ± SE)	18.0 ± 1.1	24.1 ± 1.7 ^†^	15.9 ± 1.2 ^†^	17.6 ± 1.2 ^†^	18.7 ± 1.4 ^†^

Data source: 2015 Canadian Community Health Survey—Nutrition. Yogurt consumers were defined as those individuals reporting any quantity of yogurt consumption on their day 1 of 24-h recall. ^1^ All data are weighted and bootstrapped to obtain estimates at the Canadian population level. * Significant difference across provinces at 0.05 level of significance; measured using a chi-squared test. ** Significance difference between males and females at 0.05 level of significance. ^†^ Significance difference across age groups at 0.05 level of significance.

**Table 3:** Daily energy and nutrients intake between yogurt consumers and non-consumers, all ages and by five age groups ^1^.

Nutrients	≥ 2 Years (*n* = 33,435,702)		2–5 Years (*n* = 1,222,198)		6–12 Years (*n* = 2,593,752)		13–18 Years (*n* = 2,613,677)		19–54 Years (*n* = 16,216,703)		≥55 Years (*n* = 10,789,372)	
	Yogurt Consumer (*n* = 6,748,363)	Yogurt Non-Consumer (*n* = 26687339)	Yogurt Consumer (*n* = 573,384)	Yogurt Non-Consumer (*n* = 648,814)	Yogurt Consumer (*n* = 765,650)	Yogurt Non-Consumer (*n* = 1,828,102)	Yogurt Consumer (*n* = 365,919)	Yogurt Non-Consumer (*n* = 2,247,758)	Yogurt Consumer (*n* = 2,978,009)	Yogurt non-Consumer (*n* = 13,238,694)	Yogurt Consumer (*n* = 2,065,401)	Yogurt Non-Consumer (*n* = 8,723,971)
	Mean/SE	Mean/SE	Mean/SE	Mean/SE	Mean/SE	Mean/SE	Mean/SE	Mean/SE	Mean/SE	Mean/SE	Mean/SE	Mean/SE
**Macronutrients**												
Energy (kcal)	1889.2 ± 25.9	1853.9 ± 13.9	1464.6 ± 33.2	1340.5 ± 39.9	1795.6 ± 34.7	1772 ± 26.6	2145.9 ± 70.5	2047.7 ± 27	2043.2 ± 51.2	1936.9 ± 22.6	1774.4 ± 28.8	1733.5 ± 20.5
Proteins (g)	81.1 ± 1.4	76.7 ± 0.6	60 ± 1.6	53 ± 2.3	69.3 ± 1.5	65.7 ± 1.1	88.7 ± 3.4	80.9 ± 1.3	91.5 ± 2.7	82.2 ± 1.1	74.8 ± 1.6	71.2 ± 0.89 *
Carbohydrates (g)	236.9 ± 3.2	223.6 ± 1.6 *	196.5 ± 4.7	178.8 ± 5.4	250.9 ± 5.1	244.8 ± 4.1	283.3 ± 9.9	266.4 ± 3.6	246.2 ± 6.4	226.6 ± 2.6 *	221.3 ± 4.4	206.9 ± 2.2 *
Fat (g)	68.2 ± 1.2	68.8 ± 0.7	51.1 ± 1.6	48 ± 1.7	60 ± 1.6	61.2 ± 1.1	76 ± 3.0	75.1 ± 1.4	76.6 ± 2.4	72.6 ± 1.2	62.6 ± 1.4	64.4 ± 1.1 *
Total sugar (g)	101.4 ± 1.5	87.8 ± 0.8	96.83 ± 2.3	80.3 ± 3.2	116.6 ± 3.1	107.4 ± 2.2	126.2 ± 5.3	112.6 ± 2.1	100.9 ± 2.7	86.5 ± 1.4	93.5 ± 2.2	79.8 ± 1.3 *
Cholesterol (mg)	241.6 ± 7.1	263.5 ± 3.8	182.8 ± 8.7	167.8 ± 9.1	193.7 ± 7.1	202.6 ± 6.3	270.4 ± 14.5	258.9 ± 7.1	283.4 ± 14.6	285.6 ± 6.7	210.3 ± 7.2	251 ± 5.2 *
% energy from carbohydrates	49.7 ± 0.3	48.6 ± 0.2	53.5 ± 0.6	53.4 ± 0.7	55.5 ± 0.5	54.9 ± 0.4	52.2 ± 0.7	52 ± 0.4	47.6 ± 0.6	47.3 ± 0.3	49.1 ± 0.5	48.1 ± 0.2
% energy from fats	31.3 ± 0.2	32.0 ± 0.2	30.3 ± 0.5	31.3 ± 0.5	29.1 ± 0.4	30.2 ± 0.3	31 ± 0.5	31.9 ± 0.3	32.7 ± 0.5	32.4 ± 0.3	30.5 ± 0.4	31.9 ± 0.2
% energy from proteins	17.2 ± 0.1	16.6 ± 0.1	16.2 ± 0.2	15.3 ± 0.3	15.4 ± 0.3	14.8 ± 0.2	16.7 ± 0.5	15.9 ± 0.2	18.1 ± 0.3	17 ± 0.2	16.9 ± 0.3	16.7 ± 0.1 *
MUFA (g)	24.8 ± 0.5	25.7 ± 0.3 *	17.9 ± 0.6	16.9 ± 0.6	21 ± 0.6	21.7 ± 0.4	27.4 ± 1.2	27.5 ± 0.6	28.3 ± 1.1	27.4 ± 0.5	22.7 ± 0.6	24.05 ± 0.4 *
PUFA (g)	14.2 ± 0.2	14.3 ± 0.2	8.9 ± 0.3	8.7 ± 0.3	11.2 ± 0.5	11.9 ± 0.3	14.9 ± 0.6	14.9 ± 0.3	16.1 ± 0.5	15.2 ± 0.3	13.7 ± 0.4	13.7 ± 0.3
SFA (g)	23.1 ± 0.4	22.6 ± 0.3	19.5 ± 0.7	17.9 ± 0.8	22.3 ± 0.6	22 ± 0.4 *	26.6 ± 1.2	25.9 ± 0.5 *	25.3 ± 0.8	23.5 ± 0.4	20.6 ± 0.5	20.92 ± 0.4 *
Dietary fibre (g)	18.9 ± 0.3	16.2 ± 0.2 *	13.21 ± 0.5	11.9 ± 0.5	16 ± 0.4	15.2 ± 0.3 *	18.3 ± 0.8	16.0 ± 0.3 *	20.1 ± 0.6	16.5 ± 0.2 *	19.9 ± 0.4	16.5 ± 0.26 *
**Vitamins**												
Vitamin B_12_ (mcg)	4.0 ± 0.1	3.9 ± 0.1	3.7 ± 0.1	3.2 ± 0.1	3.6 ± 0.1	3.4 ± 0.1	4.7 ± 0.3	4.29 ± 0.2	4.3 ± 0.2	4.1 ± 0.1	3.8 ± 0.1	3.8 ± 0.1
Vitamin B_6_ (mg)	1.7 ± 0.03	1.6 ± 0.01	1.2 ± 0.03	1.1 ± 0.05	1.4 ± 0.04	1.4 ± 0.03	1.7 ± 0.1	1.6 ± 0.03	1.9 ± 0.1	1.7 ± 0.03	1.6 ± 0.04	1.5 ± 0.02
Vitamin A RAE (mcg)	682.2 ± 18.3	620.2 ± 9.6	575.3 ± 26.6	575.7 ± 34.8	583.2 ± 21	569 ± 14.1	655.2 ± 30.1	646.7 ± 24.6	721.3 ± 34.3	616.3 ± 15.4	696.9 ± 33.8	633.4 ± 15.1
Riboflavin (mg)	2.1 ± 0.03	1.8 ± 0.01 *	1.8 ± 0.1	1.5 ± 0.1	1.8 ± 0.04	1.7 ± 0.03	2.2 ± 0.1	1.9 ± 0.03	2.2 ± 0.1	1.9 ± 0.02	2.0 ± 0.04	1.8 ± 0.02
Thiamin (mg)	1.61 ± 0.03	1.6 ± 0.01 *	1.2 ± 0.04	1.2 ± 0.05	1.6 ± 0.04	1.6 ± 0.03	1.9 ± 0.1	1.8 ± 0.03	1.7 ± 0.1	1.6 ± 0.02	1.5 ± 0.04	1.5 ± 0.02
Vitamin C (mg)	115.2 ± 3.1	97.3 ± 1.6 *	106.4 ± 5.9	103.8 ± 6.1	123.2 ± 5.5	116.4 ± 3.6	113.5 ± 7.1	114.2 ± 3.5	118.8 ± 6	96.6 ± 2.8 *	109.8 ± 4.6	89.4 ± 2.3 *
Folate DFE (mcg)	442.6 ± 8.5	436.4 ± 3.7 *	319.3 ± 13.1	321.7 ± 11.5	403.6 ± 12.9	434.3 ± 10.3	499.4 ± 17.2	486.4 ± 10.4	496.7 ± 17.8	460.6 ± 6.3 *	403.2 ± 9.9	395.7 ± 5.6
Vitamin D (mcg)	5.4 ± 0.1	4.7 ± 0.1 *	6.1 ± 0.2	5.7 ± 0.3	5.5 ± 0.2	5.1 ± 0.1	6.6 ± 0.3	5.4 ± 0.1	5.3 ± 0.2	4.4 ± 0.1 *	5.1 ± 0.2	4.8 ± 0.1
Folic Acid (mcg)	110.8 ± 3.2	116.9 ± 1.5	90.5 ± 6.4	93.4 ± 4.9	122.2 ± 5.4	137.2 ± 4	152.8 ±7.8	150.1 ± 4.4	121.2 ± 6.4	120.6 ± 2.6	89.9 ± 4.1	100.2 ± 2.1
Niacin NE (mg)	38.2 ± 0.7	38 ± 0.3	25.9 ± 0.7	23.8 ± 0.0	31.2 ± 0.7	31.3 ± 0.5	40.7 ± 1.7	39.4 ± 0.7	43.9 ± 1.5	40.9 ± 0.6	35.5 ± 0.9	35.6 ± 0.4 *
**Minerals**												
Potassium (mg)	2881.6 ± 39.9	2560.5 ± 18.5 *	2227.2 ± 59.7	1988.1 ± 78.4	2508.9 ± 50.1	2276.9 ± 36.1	2821.8 ± 104.4	2512.3 ± 36.2	3081.3 ± 76.8	2617.7 ± 30.3 *	2923.9 ± 56.5	2588.3 ± 30.6
Iron (mg)	12.5 ± 0.2	12.2 ± 0.1 *	8.9 ± 0.3	8.6 ± 0.3	11.9 ± 0.3	12.2 ± 0.2	14.3 ± 0.7	13.3 ± 0.2	13.6 ± 0.4	12.4 ± 0.1 *	11.8 ± 0.3	11.7 ± 0.1
Magnesium (mg)	322.1 ± 5.2	291.1 ± 2.2 *	221.9 ± 6.2	202.1 ± 6.4	258.7 ± 4.8	243.6 ± 4.1	319.5 ± 14.3	282.6 ± 4.5	356.4 ± 10	303.1 ± 3.5 *	324.1 ± 6.4	291.7 ± 3.8
Sodium (mg)	2588.3 ± 47	2720.6 ± 25.3	1902.4 ± 55.9	1825.1 ± 78.2	2473.4 ± 63.8	2498.2 ± 46.1	2929.3 ± 99.3	2947.6 ± 47.6	2816.6 ± 93.2	2881 ± 42.8	2431.6 ± 53.9	2531.9 ± 35
Calcium (mg)	977.3 ± 16.6	767.7 ± 8.4 *	1007.6 ± 30.9	847.8 ± 33.6	1018.7 ± 25.5	876.7 ± 17.7	1174.1 ± 50.2	937.1 ± 18.4	994.1 ± 30.3	770.2 ± 13.9 *	894.5 ± 23.7	691.4 ± 10.3 *
Zinc (mg)	10.4 ± 0.1	10.2 ± 0.1	7.6 ± 0.2	6.9 ± 0.3	8.9 ± 0.2	8.6 ± 0.2	11.5 ± 0.6	10.6 ± 0.2	11.4 ± 0.4	10.7 ± 0.2	9.8 ± 0.2	9.8 ± 0.2

Data source: 2015 Canadian Community Health Survey—Nutrition. Yogurt consumers were defined as those individuals reporting any quantity of yogurt consumption on their day 1 of 24-h recall. ^1^ All data are weighted and bootstrapped to obtain estimates at the Canadian population level. The regression model was adjusted for significant differences between Yogurt consumers and non-consumers in age, sex, smoking, ethnicity, education, marital status, food security, overweight/obesity, immigrant and daily energy intake. NE = niacin equivalents; DFE = dietary folate equivalents; RAE = retinol activity equivalents. * Intake difference between Yogurt consumers and non-consumers for all ages and each age group at 0.05 level of significance.

**Table 4:** Percent contribution from yogurt to daily energy and nutrient intake (yogurt consumers) ^1^.

Nutrients	≥2 Years (*n* = 674,836)	2–5 Years (*n* = 573,384)	6–12 Years (*n* = 765,650)	13–18 Years (*n* = 365,919)	19–54 Years (*n*=2,978,009)	≥55 Years (*n* = 2,065,401)
	% Contribution from Yogurt to Daily Nutrient Intake	% Contribution from Yogurt to Daily Nutrient Intake	% Contribution from Yogurt to Daily Nutrient Intake	% Contribution from Yogurt to Daily Nutrient Intake	% Contribution from Yogurt to Daily Nutrient Intake	% Contribution from Yogurt to Daily Nutrient Intake
	% (SE)	% (SE)	% (SE)	% (SE)	% (SE)	% (SE)
Energy (%)	5.7 ± 0.1	6.8 ± 0.3	5.7 ± 0.3	5.1 ± 0.3	5.4 ± 0.2	5.7 ± 0.2
Carbohydrates (%)	6.3 ± 0.2	7.3 ± 0.3	6.0 ± 0.3	5.4 ± 0.4	6.3 ± 0.3	6.4 ± 0.3
Dietary fibre (%)	1.0 ± 0.1	0.9 ± 0.3	0.5 ± 0.2	1.5 ± 0.4	1.4 ± 0.3	0.6 ± 0.1 *
Total sugars (%)	13.6 ± 0.4	13.4 ± 0.6	11.6 ± 0.5	11.6 ± 0.9	13.9 ± 0.7	14.3 ± 0.6 *
Fat (%)	3.9 ± 0.1	5.2 ± 0.3	4.4 ± 0.3	3.9 ± 0.3	3.7 ± 0.3	3.7 ± 0.2 *
Saturated fatty acids (%)	7.0 ± 0.3	8.6 ± 0.4	7.6 ± 0.5	6.9 ± 0.5	6.8 ± 0.5	6.7 ± 0.4
Monounsaturated fatty acids (%)	3.0 ± 0.1	4.0 ± 0.2	3.4 ± 0.2	3.0 ± 0.3	2.7 ± 0.2	2.8 ± 0.2 *
Polyunsaturated fatty acids (%)	1.1 ± 0.1	1.5 ± 0.2	1.3 ± 0.2	1.0 ± 0.1	0.9 ± 0.1	1.2 ± 0.2
Cholesterol (%)	6.7 ± 0.3	7.6 ± 0.5	6.9 ± 0.5	6.3 ± 0.6	6.3 ± 0.5	7.0 ± 0.4
Protein (%)	8.7 ± 0.2	9.3 ± 0.6	7.7 ± 0.3	7.6 ± 0.5	8.3 ± 0.4	9.6 ± 0.4 *
Vitamin A RAE (%)	5.2 ± 0.2	4.3 ± 0.3	4.3 ± 0.3	5.1 ± 0.5	5.3 ± 0.4	5.6 ± 0.4 *
Vitamin D (%)	16.2 ± 0.6	13.9 ± 0.9	15.1 ± 1.0	14.0 ± 1.1	16.3 ± 1.1	17.4 ± 1.1 *
Vitamin C (%)	1.8 ± 0.2	1.1 ± 0.2	1.3 ± 0.3	1.8 ± 0.4	1.7 ± 0.2	2.4 ± 0.5 *
Thiamine (%)	2.8 ± 0.1	2.2 ± 0.2	1.7 ± 0.1	2.2 ± 0.2	2.9 ± 0.2	3.4 ± 0.2 *
Riboflavin (%)	11.8 ± 0.3	12.0 ± 0.5	11.9 ± 0.5	11.2 ± 0.6	11.3 ± 0.5	12.5 ± 0.4
Niacin NE (%)	3.9 ± 0.1	3.7 ± 0.3	2.9 ± 0.2	3.5 ± 0.3	3.8 ± 0.2	4.6 ± 0.2 *
Vitamin B_6_ (%)	2.9 ± 0.1	2.5 ± 0.3	2.1 ± 0.1	2.7 ± 0.2	2.7 ± 0.2	3.6 ± 0.2 *
Vitamin B_12_ (%)	13.7 ± 0.4	11.8 ± 0.7	11.8 ± 0.6	11.7 ± 0.8	13.9 ± 0.7	15.0 ± 0.6 *
Folate DFE (%)	1.9 ± 0.1	1.9 ± 0.2	1.6 ± 0.1	1.2 ± 0.1	1.7 ± 0.1	2.3 ± 0.2 *
Calcium (%)	18.3 ± 0.4	16.3 ± 1.0	14.9 ± 0.6	16.8 ± 1.1	19.1 ± 0.8	19.1 ± 0.6 *
Magnesium (%)	4.8 ± 0.1	5.9 ± 0.2	5.4 ± 0.2	4.8 ± 0.3	4.4 ± 0.2	4.8 ± 0.2 *
Iron (%)	1.4 ± 0.1	1.6 ± 0.1	1.4 ± 0.1	1.1 ± 0.1	1.2 ± 0.1	1.7 ± 0.1
Zinc (%)	5.5 ± 0.1	6.2 ± 0.3	5.9 ± 0.3	4.7 ± 0.4	4.9 ± 0.3	6.2 ± 0.2
Sodium (%)	2.8 ± 0.1	3.1 ± 0.1	2.5 ± 0.1	2.3 ± 0.1	2.8 ± 0.2	3.0 ± 0.2 *
Potassium (%)	7.8 ± 0.2	8.8 ± 0.3	8.5 ± 0.3	8.1 ± 0.4	7.3 ± 0.3	7.8 ± 0.3

Data source: 2015 Canadian Community Health Survey—Nutrition. ^1^ All data are weighted and bootstrapped to obtain estimates at the Canadian population level. Yogurt consumers were defined as those individuals reporting any quantity of yogurt consumption on their day 1 of 24-h recall. Data are shown as unadjusted means (SE) and are weighted to the Canadian population NE = niacin equivalents; DFE = dietary folate equivalents; RAE = retinol activity equivalents. * Percentage difference of contributions within age groups for contribution to daily nutrients at 0.05 level of significance.

**Table 5:** The NRF 9.3 score of yogurt consumers and non-consumers across Canada (all ages, 5 age groups) and regions (all ages and 2 age groups) ^1^.

Age	Yogurt Consumer	Yogurt Non-Consumer
	NRF 9.3 Score (Mean, SE)	NRF 9.3 Score (Mean, SE)
	**National (*n* = 33,435,702)**	
≥ 2 years (All Canadians)	*n* = 6,748,363	*n* = 26,687,339
	558.7 ± 3.2	507.2 ± 2.1 *
2–5 years	*n* = 573,384	*n* = 648,814
	563.2 ± 7.6	561.3 ± 7.0
6–12 years	*n* = 765,650	*n* = 1,828,102
	537.9 ± 6.4	514.8 ± 4.3
13–18 years	*n* = 365,919	*n* = 2,247,758
	515.4 ± 9.7	480.8 ± 4.0 *
19–54 years	*n* = 2,978,009	*n* = 13,238,694
	554.6 ± 5.6	493.9 ± 3.3 *
≥ 55 years	*n* = 2,065,401	*n* = 8,723,971
	578.8 ± 5.1	528.7 ± 3.3 *
	**Atlantic (*n* = 2,223,781)**	
≥ 2 years (All Atlantic residents)	*n* = 409,046	*n* = 1,814,735
	550.8 ± 6.4	486.3 ± 3.5 *
2–18 years	*n* = 79,891	*n* = 313,935
	544.1 ± 7.2	480.9 4.8 *
≥ 19 years	*n* = 329,155	*n* = 1,500,800
	552.4 ± 7.6	487.3 4.2 *
	**Quebec (*n* = 7,811,646)**	
≥ 2 years (All Quebec residents)	*n* = 1,988,464	*n* = 5,823,182
	556.7 ± 6.8	505.9 ± 3.9 *
2–18 years	*n* = 447,992	*n* = 969,507
	539.8 ± 7.1	515.6 ± 5.4 *
≥ 19 years	*n* = 1,540,472	*n* = 4,853,675
	561.6 ± 8.4	504 ± 4.4 *
	**Ontario (*n* = 12,994,037)**	
≥ 2 years (All Ontario residents)	*n* = 2,327,589	*n* = 10,666,448
	561.7 ± 5.3	514.4 ± 3.9 *
2–18 years	*n* = 665,855	*n* = 1,895,760
	544.8 ± 8.4	509.4 ± 5.9 *
≥ 19 years	*n* = 1,661,734	*n* = 8,770,688
	568.5 ± 6.7	515.4 ± 4.7 *
	**Prairie (*n* = 6,012,754)**	
≥ 2 years (All Prairie residents)	*n* = 1,142,808	*n* = 4,869,946
	546.2 ± 7.5	493.7 ± 4.1 *
2–18 years	*n* = 304,887	*n* = 967,132
	529.4 ± 8.4	487.4 ± 4.3 *
≥ 19 years	*n* = 837,921	*n* = 3,902,814
	552.3 ± 10.1	495.2 ± 4.9 *
	**British Columbia (*n* = 4,393,484)**	
≥ 2 years (All BC residents)	*n* = 880,456	*n* = 3,513,028
	575.2 ± 8.8	517.3 ± 5.4 *
2–18 years	*n* = 206,328	*n* = 578,340
	552.1 ± 9.8	515.1 ± 6.6 *
≥ 19 years	*n* = 674,128	*n* = 2,934,688
	582.2 ± 10.9	517.7 ± 6.3 *

Data source: 2015 Canadian Community Health Survey—Nutrition. Yogurt consumers were defined as those individuals reporting any quantity of yogurt consumption on their day 1 of 24-h recall. ^1^ All data are weighted and bootstrapped to obtain estimates at the Canadian population level. The NRF 9.3 score had been calculated using twelve nutrients as follows: protein, fibre, vitamin A, vitamin D, vitamin C, calcium, iron, magnesium, potassium, saturated fat, total sugar and sodium [11]. This model had been adjusted by age, sex, smoking, ethnicity, education, marital status, food security, overweight/obesity, immigrant and daily energy intake for significant differences between yogurt consumers and non-consumers. * Significant differences of the Nutrient Rich Foods index (NRF 9.3) score between yogurt consumers and non-consumers.

## Abbreviations

Polyunsaturated fatty acids (PUFA), Monounsaturated fatty acids (MUFA), Saturated fatty acids (SFA), Canadian Community Health Survey (CCHS), Nutrient-rich foods index (NRF), Body mass index (BMI), British Columbia (BC), DV (Daily Value).
